# Characterization of Possible α-Glucosidase Inhibitors from *Trigonella stellata* Extract Using LC–MS and In Silico Molecular Docking

**DOI:** 10.3390/plants11020208

**Published:** 2022-01-14

**Authors:** Ahlam Elwekeel, Dalia El Amir, Enas I. A. Mohamed, Elham Amin, Marwa H. A. Hassan, Mohamed A. Zaki

**Affiliations:** 1 Department of Pharmacognosy, Faculty of Pharmacy, Beni-Suef University, Beni-Suef 62514, Egypt; Ahlam.hassanain@pharm.bsu.edu.eg (A.E.); Dalia.elamir@pharm.bsu.edu.eg (D.E.A.); enas.mohamed@pharm.bsu.edu.eg (E.I.A.M.); elham_bns@yahoo.com (E.A.); 2 Department of Medicinal Chemistry and Pharmacognosy, College of Pharmacy, Qassim University, Buraidah 51452, Saudi Arabia

**Keywords:** *Trigonella stellata*, LC-HRESIMS, molecular docking, α-glucosidase inhibition, graecumoside A, fenugreekine

## Abstract

The current study accentuates the significance of performing the multiplex approach of LC-HRESIMS, biological activity, and docking studies in drug discovery, taking into consideration a review of the literature. In this regard, the investigation of antioxidant and cytotoxic activities of *Trigonella stellata* collected from the Egyptian desert revealed a significant antioxidant capacity using DPPH with IC_50_ = 656.9 µg/mL and a moderate cytotoxicity against HepG2, MCF7, and CACO2, with IC_50_ values of 53.3, 48.3, and 55.8 µg/mL, respectively. The evaluation of total phenolic and flavonoid contents resulted in 32.8 mg GAE/g calculated as gallic acid equivalent and 5.6 mg RE/g calculated as rutin equivalent, respectively. Chemical profiling of *T. stellata* extract, using LC-HRESIMS analysis, revealed the presence of 15 metabolites, among which eleven compounds were detected for the first time in this species. Interestingly, in vitro testing of the antidiabetic activity of the alcoholic extract noted an α-glucosidase enzyme inhibitory activity (IC_50_ = 559.4 µg/mL) better than that of the standard Acarbose (IC_50_ = 799.9 µg/mL), in addition to a moderate inhibition of the α-amylase enzyme (IC_50_ = 0.77 µg/mL) compared to Acarbose (IC_50_ = 0.21 µg/mL). α-Glucosidase inhibition was also virtualized by binding interactions through the molecular docking study, presenting a high binding activity of six flavonoid glycosides, as well as the diterpenoid compound graecumoside A and the alkaloid fenugreekine. Taken together, the conglomeration of LC-HRESIMS, antidiabetic activity, and molecular docking studies shed light on *T. stellata* as a promising antidiabetic herb.

## 1. Introduction

Diabetes mellitus (DM) is a chronic disorder marked by raised blood glucose levels due to disruption in the secretion of insulin, or its utilization or both [1]. It is highly prevalent and, if not properly managed, brings about serious complications; therefore, it is a major medical issue worldwide [2,3,4]. Antidiabetic drugs could control blood glucose levels via several mechanisms, e.g., induction of pancreatic β-cells to produce more insulin, enhancing sensitivity of insulin receptors, and inhibition of carbohydrates-digesting enzymes such as α-glucosidase and α-amylase enzymes [5]. In vitro testing of the inhibitory activity of plant extracts and/or pure isolated phytoconstituents against carbohydrates-metabolizing enzymes represents a preliminary step in antidiabetic activity screening programs. In this context, significant inhibitory activities of several plant constituents, e.g., vitexin, isovitexin, and kaempferol, against α-glucosidase and α-amylase enzymes were previously reported [6,7]. Moreover, a molecular docking study of the activity of kaempferol and diosgenin against α-glucosidase and α-amylase was also reported [8,9]. The development of new antidiabetic medicines from natural origin appears as an attractive approach due to safety or cost concerns associated with most of the current medications [2]. However, the complexity of the metabolic pattern of natural sources represents a serious issue that controls the performance of natural product screening programs.

Currently, dereplication plays a crucial role in the drug discovery process as it is a fast and reliable approach, allowing scientists to focus on novel bioactive natural products. It helps to confront the significant challenges in drug discovery procedures, including the difficulty to interpret biological activities of crude extracts due to the presence of numerous metabolites with a great variation in their physicochemical properties and abundance levels [10,11,12].

Computational methods, especially molecular docking, are widely used in drug discovery and drug design processes, where compounds (ligands) are “docked” into the binding site of the specific target (receptor) and then the binding affinity is scored depending on the complementarity of the docked compounds to the binding site [13]. Generally, there are two goals of a docking study: structural drug design and investigation of specific activity, and studying its mechanism at the molecular level [14]. A panel of interactions between a ligand and receptor include hydrogen bond, electrostatic interaction, and hydrophobic contacts [15]. Molecular docking is highly beneficial when applied to analyze and investigate experimental results and provides instructions for future research.

Desert regions represent wide areas in Egypt in which variable species of wild plants grow. The harsh conditions, such as the salinity and scarcity of water, direct these plants to synthesize variable secondary metabolites as a survival mechanism in such habitats. These metabolites impart medicinal value to wild plants and explain the numerous folk uses of these plants. Several species of wild plants growing in Egypt were proven to exhibit significant anti-inflammatory, antimicrobial, anticancer, antidiabetic, etc. activities [16]. 

*Trigonella stellata* is a wild plant growing in the Egyptian desert, belonging to the genus *Trigonella* (family Leguminosae), that is distributed mainly in the Mediterranean region. The genus *Trigonella* encompasses several species, out of which *T. foenum-graecum* is used traditionally in many areas of the world as a carminative, tonic, and aphrodisiac [17], in addition to its antidiabetic and antihyperlipidemic effects [18]. Numerous phytochemicals were detected in this genus such as saponins, alkaloids, and flavonoids [19]. Previous studies on *T. stellata* reported the isolation of three new isoflavans identified as (3S,4R)-4,2′,4′-trihydroxy-7-methoxyisoflavan, (3R,4S)-4,2′,4′-trihydroxy-7-methoxy-4′-*O*-β-D-glucopyranosyl isoflavane, and (2S,3R,4R)-4,2′,4′-trihydroxy-2,7-dimethoxyisoflavan, in addition to other known phytochemicals [20]. Another study reported the isolation of caffeic acid from *T. stellata* and evaluated its antiosteoporosis activity [21]. The antidiabetic activity of the alcoholic extract of *T. stellata* was previously assessed through evaluation of the activation of PPARα and PPARγ in human hepatoma (HepG2) cells [20]. In addition, the protective effect of *T. stellata* extract against adverse effects caused by diabetes was evaluated [22].

In spite of various clinical developments, cancer still one of the main causes of death worldwide. By 2030, twelve million deaths have been predicted to occur due to cancer by the World Health Organization (WHO). Most anticancer drugs are accompanied by side-effects and nonspecificity. Hence, there is a great interest in finding safe, selective, and effective anticancer drugs [23,24]. In this regard, human cancer cell lines are considered essential models to evaluate the efficacy of anticancer agents, with breast, liver, colon, and prostate cell lines being the four major types of cell lines tested [25,26].

Herein, our approach taken together is as follows: a review of the literature, biological activity, chemical profiling, and computational studies to point out *T. stellata* as a promising bioactive candidate. Acting on this premise, we examined the antioxidant, cytotoxic, and antidiabetic potential of the plant, followed by identification of phytochemicals in *T. stellata* alcoholic extract using liquid chromatography-high resolution electrospray ionization mass spectrometry (LC-HRESIMS), in addition to the estimation of total phenolic (TPC) and flavonoid (TFC) contents. Furthermore, we explored the compounds expected to be responsible for the antidiabetic activity through molecular docking of the identified compounds toward the α-glucosidase enzyme. 

## 2. Results and Discussion

### 2.1. Total Phenolic (TPC) and Total Flavonoid (TFC) Contents

Phenolics are a major class of active compounds detected in plants, which exhibit many activities such as antioxidant, antitumor, anti-inflammatory, and antidiabetic [27]. Flavonoids are among the most abundant phenolic compounds in plants. The phenolics and flavonoids contents of plant extracts are usually determined using Folin–Ciocalteu [28] and AlCl_3_ reagent [29] methods, respectively. The current study is the first report for the estimation of the TPC and TFC of *T. stellata*, where their TPC was estimated as 32.8 ± 1.57 mg GAE/g and their TFC was 5.6 ± 0.14 mg RE/g. However, several previous studies discussed the contents of TP and TF in other *Trigonella* species, e.g., *T. spruneriana* [30] and *T. foenum graecum* leaves [31], seeds [32], and explant [33]. The phenolic compounds represent an important class of secondary metabolites with pronounced antioxidant properties. The presence of such metabolites, in a considerably good amount, suggests the powerful antioxidant potential of the tested extracts [34]. Accordingly, *T. stellata* might be a promising candidate for variable biological activities screening.

### 2.2. Biological Activities

#### 2.2.1. Antioxidant Activity

DPPH is a stable free radical commonly used for determining the antioxidant capacity of plant extracts and is characterized by its violet color, which can be reduced via accepting a hydrogen atom from antioxidants [35]. The antioxidant capacity of *T. stellata* alcoholic extract was investigated through measuring its ability to scavenge DPPH radicals. Results revealed a significant antioxidant activity of *T. stellata* with IC_50_ = 656.9 ± 9.64 µg/mL, compared to ascorbic acid (IC_50_ = 163.06 ± 1.4 µg/mL). These results came in great accordance with the recorded TPC and TFC of the plant. This means that the observed antioxidant activity of *T. stellata* extract could be attributable to its content of phenolic constituents. As previously mentioned, the current study is the first report for the analysis of the phytochemical content and investigation of the antioxidant capability of *T. stellata*; nevertheless, the antioxidant potential of several *Trigonella* species was previously reported [30,31,32,33]. Among these reports, one study tested the antioxidant activity of *T. foenum graecum* using different in vitro and ex vivo methods and concluded potent activity for the seed extract [36].

#### 2.2.2. Antidiabetic Activity

Numerous medicinal plants have been reported for antidiabetic activity, modulated via different mechanisms, e.g., increased pancreatic secretion of insulin, inhibition of glucose absorption, and enhanced glucose uptake by muscles and adipose tissue. Furthermore, the inhibition of carbohydrates-metabolizing enzymes, e.g., α-amylase and α-glucosidase, is one of the important approaches approved for the management of diabetes. These enzymes are responsible for the breakdown of oligosaccharides and disaccharides into monosaccharides that can be absorbed through the intestinal mucosa into the bloodstream. The inhibition of these enzymes results in a delayed carbohydrate digestion with a subsequent decrease in the blood glucose level, followed by decreased insulin secretion [37]. Previous research on *T. stellata* concluded that the ethyl acetate extract of aerial parts of the plant exhibited antidiabetic activity through the stimulation of PPARα and PPARγ receptor activity [20]. Furthermore, another study reported the protective effect of *T. stellata* extract against diabetes complications via investigation of the changes in phase I and II drug-metabolizing enzyme activities and the protein expression of cytochrome P450 [22]. Herein, the inhibitory effect of *T. stellata* extract against α-glucosidase and α-amylase enzymes was investigated. The alcoholic extract of *T. stellata* showed good inhibitory activity against the α-glucosidase enzyme (IC_50_ = 559.37 ± 25.67 µg/mL) and moderate inhibitory activity against the α-amylase enzyme (IC_50_ = 0.773 ± 0.05 µg/mL), when compared to Acarbose^®^ (IC_50_ = 799.92 ± 36.7 µg/mL and 0.210 ± 0.01 µg/mL against α-glucosidase and α-amylase, respectively). A literature survey highlighted similar α-glucosidase and α-amylase inhibitory activities of *T. foenum graecum* extract as one of the proposed mechanisms for its pronounced hypoglycemic effect [38,39]. The current results suggest that the observed inhibitory effect against carbohydrates-metabolizing enzymes could be an additional mechanism for the antidiabetic activity of *T. stellata*, hence indicating that the antidiabetic activity of this plant may occur through multi-target mechanisms.

#### 2.2.3. Cytotoxic Activity

Nowadays, plenty of research projects are directed toward the screening of natural sources for possible anticancer activity [39]. In this regard, the alcoholic extract of *T. stellata* was evaluated in vitro for cytotoxic activity against three carcinoma cell lines: liver carcinoma (HepG2), breast carcinoma (MCF7), and intestinal carcinoma (CACO2) cell lines. A previous test of the cytotoxic activity of the alcoholic extract of *T. stellata*, using brine shrimp bioassay, indicated a potent activity with an IC_50_ value of 2.6 µg/mL [40]. Herein, the current results revealed moderate activity of the alcoholic extract against the three tested cell lines with IC_50_ values of 53.3, 48.3, and 55.8 µg/mL against HepG2, MCF7, and CACO2, respectively, as compared to the reference drug Doxorubicin^®^ (IC_50_ values of 3.8, 4.2, and 3.4 µg/mL, respectively). Similarly, the seeds extract of *T. foenum graecum* was reported to induce apoptosis in HepG2 cells [41]. In conclusion, the observed cytotoxic activity of *T. stellata* extract might be related to the recorded antioxidant activity of the plant, which in turn could be caused by its high content of phenolic compounds.

### 2.3. Chemical Profiling

**Figure 1:**
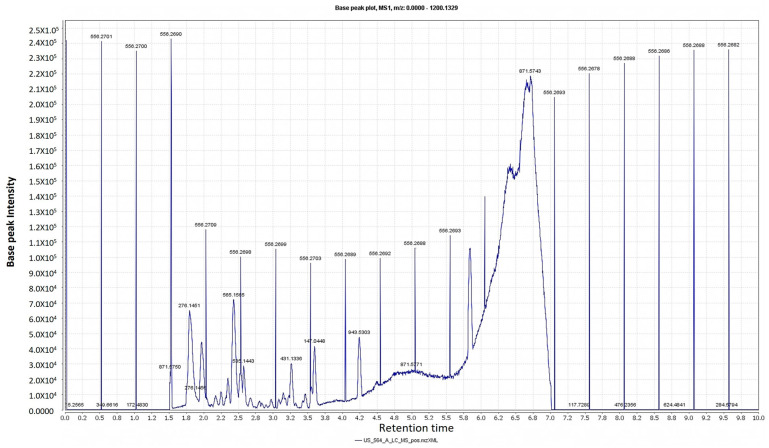
LC-HRESIMS chromatogram of the dereplicated metabolites of *Trigonella stellata* (positive).

**Figure 2:**
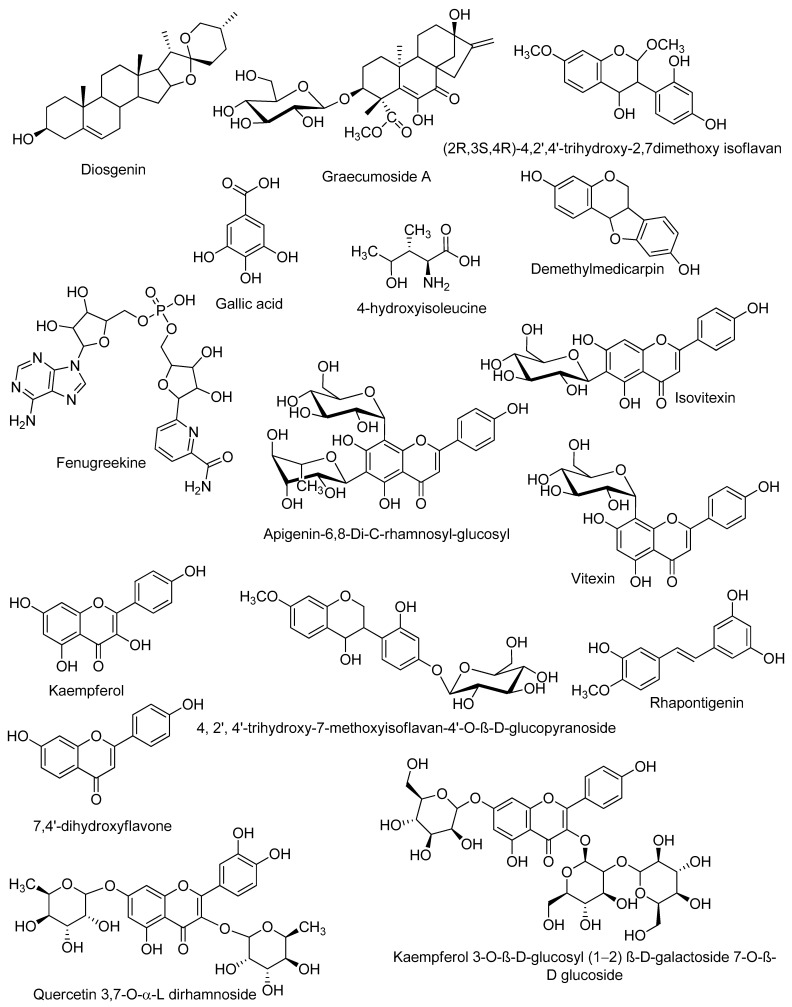
Structures of the dereplicated compounds from *Trigonella stellata* methanolic extract by LC-HRESIMS.

Classical purification and isolation techniques are costly, time-consuming, and may lead to the isolation of previously identified compounds. The dereplication technique is an efficient way to avoid the unnecessary purification work; hence, it is cost-effective, saves time and effort, and enables the targeted discovery of new bioactive natural products. Chemical profiling of the alcoholic extract of *T. stellata* (Table 1, Figure 1 and Figure 2) led to the characterization of eleven compounds for the first time from *T. stellata* extract. The molecular ion peaks at *m*/*z* 538.8999, 147.0239, 256.1580, 663.3963, 414.2121, 258.1368, and 171.0821, corresponding to the suggested molecular formulas C_27_H_38_O_11_, C_6_H_13_NO_3_, C_15_H_12_O_4_, C_21_H_27_N_7_O_14_P_2_, C_27_H_42_O_3_, C_15_H_14_O_4_, and C_7_H_6_O_5_, respectively, were detected. These peaks were dereplicated as the kaurene diterpenoid glycoside (graecumoside A) **1** [42], amino acid (4-hydroxyisoleucine) **2** [43], pterocarpan (demethylmedicarpin) **3** [44], alkaloid (fenugreekine) **4** [45], phytosteroid sapogenin (diosgenin) **5** [46], stilbene (rhapontigenin) **6** [47], and gallic acid **7** [45], which were previously isolated from *T. foenum-graecum*. Meanwhile, the other four molecular ion peaks at *m*/*z* 285.2079, 771.6169, 432.2447, and 577.2966, consistent with the molecular formulas C_15_H_10_O_6_, C_33_H_41_O_23_, C_21_H_20_O_10_, and C_21_H_30_O_14_, respectively, were dereplicated as the flavonol (kaempferol) **8** [45], flavonol glycosides (kaempferol 3-O-β-D-glucosyl (1 → 2) β-D-galactoside 7-O-β-D glucoside) **9** [48], flavone glycosides isovitexin or vitexin **10** [45], and apigenin-6,8-di-C-rhamnosyl-glucosyl **11** [49]. Other molecular ion peaks at *m*/*z* 450.3261, 318.2840, 254.1596, and 593.9521 corresponding to the suggested molecular formulas C_22_H_26_O_10_, C_17_H_18_O_6_, C_15_H_10_O_4_, and C_27_H_30_O_15_ were dereplicated as the flavonoids: 4, 2′, 4′-trihydroxy-7-methoxyisoflavan-4′-O-β-D-glucopyranoside **12**, (2R,3S,4R)-4,2′,4′-trihydroxy-2,7dimethoxy isoflavan **13**, 7,4′-dihydroxyflavone **14**, and quercetin 3,7-O-α-L dirhamnoside **15**, which were previously reported from *T. stellata* [20]. 

### 2.4. Molecular Docking

In an attempt to understand the observed antidiabetic activity of *T. stellata* extract against the α-glucosidase enzyme, a docking study was performed to explore the compounds expected to be responsible for the enzyme inhibitory activity and their binding modes to the human lysosomal acid α-glucosidase enzyme. The catalytic site of the lysosomal human α-glucosidase enzyme consists mainly of acidic residues (including Asp282, Asp404, Asp518, Asp616, and Asp645) and basic residues (Arg600, Arg672, and His674) [50]. The molecular docking study of compounds identified in *T. stellata* extract, toward α-glucosidase, in terms of hydrogen and hydrophobic interactions with key amino acids, revealed a high binding activity of some of the tested compounds. These compounds include quercetin-3,7-*O*-α-L di-rhamnoside, kaempferol 3-*O*-β-D-glucosyl (1 → 2) β-D-galactoside 7-*O*-β-D glucoside, apigenin-6,8-di-*C*-rhamnosyl-glucosyl, vitexin, isovitexin, 4,2′,4′-trihydroxy-7-methoxyisoflavan-4′-*O*-β-D-glucopyranoside, graecumoside A, and fenugreekine.

**Figure 3:**
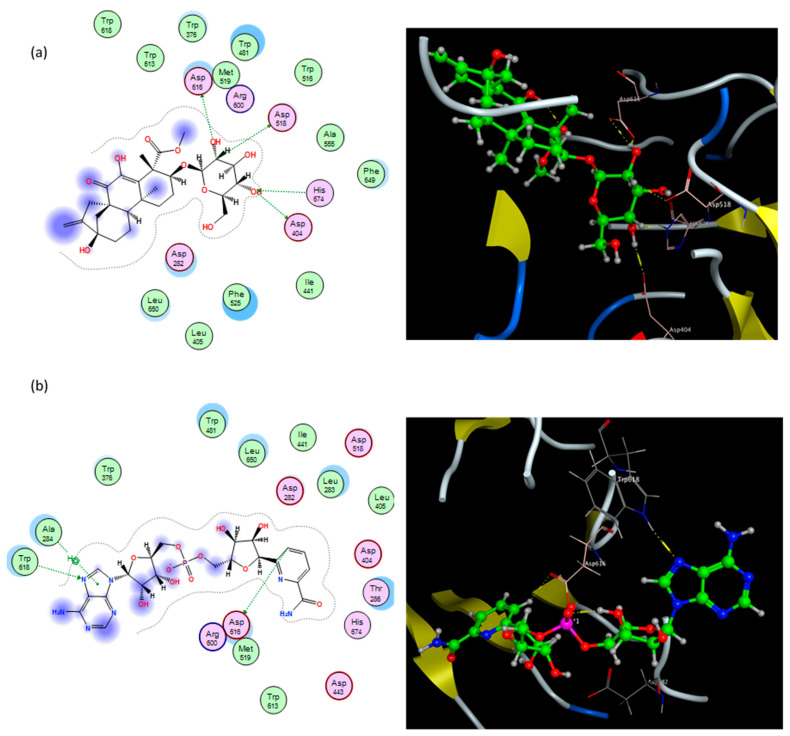
Binding modes of graecumoside A (**a**) and fenugreekine (**b**) into human α-glucosidase active site. In the 3D view (right side), ligands are depicted in the ball and stick model. The residues potentially interacting with the ligands are shown in labeled tube models; the dotted line indicates the formation of hydrogen bonds with amino acid residues. In the 2D view (left side), pink circles with red and blue borders indicate polar acidic and basic amino acids, respectively, and green circles indicate nonpolar amino acids.

Interestingly, the diterpene graecumoside A and the alkaloid fenugreekine revealed good binding interactions with the α-glucosidase enzyme. As can be noticed in Figure 3a, the diterpene graecumoside A is fitted well in the active site of the receptor, and it constructed four hydrogen bond interactions: with Asp404, Asp518, Asp616, and His674, in addition to two hydrophobic interactions with Trp481 and two hydrophobic interactions with Phe525. It is noteworthy that this is the first simulated molecular docking investigation of graecumoside A into an antidiabetic target where binding energy and key interactions of graecumoside A with α-glucosidase are promising, and it may provide guidance for a more detailed investigation for its antidiabetic activity. On the other hand, the alkaloid fenugreekine exhibited two hydrogen bond interactions with Asp616 and Trp618, a *π*–hydrogen interaction with Ala284, in addition to two hydrophobic contacts with Ile441 and Trp516 that helped fenugreekine to anchor in the active site (Figure 3b). 

**Figure 4:**
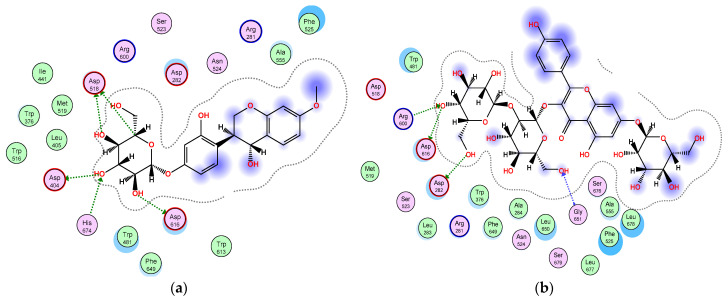

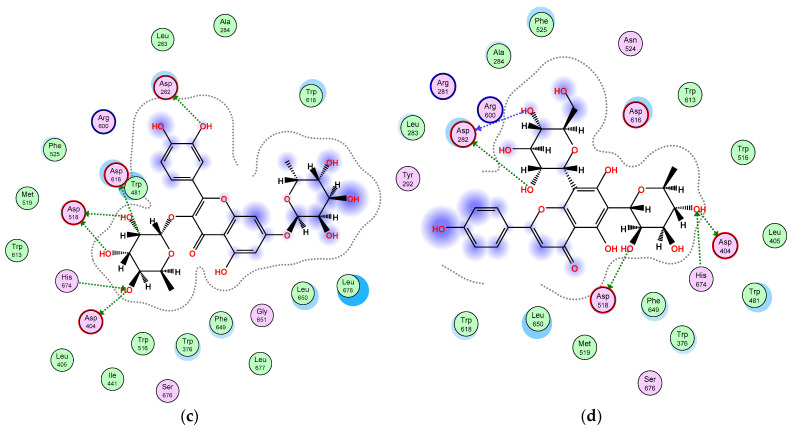
2D diagram of the binding interactions of (**a**) 4,2′,4′-trihydroxy-7-methoxyisoflavan-4′-O-β-D-glucopyranoside, (**b**) kaempferol 3-*O*-β-D-glucosyl (1 → 2) β-D-galactoside 7-*O*-β-D-glucoside, (**c**) quercetin 3,7-*O*-α-L dirhamnoside, and (**d**) apigenin-6,8-di-*C*-rhamnosyl-glucosyl with the active site residues of the α-glucosidase receptor. Pink circles with red and blue borders indicate polar acidic and basic amino acids, respectively, and green circles indicate nonpolar amino acids.

Furthermore, among the docked compounds, the six flavonoid glycosides: quercetin-3,7-*O*-α-L di-rhamnoside, kaempferol 3-*O*-β-D-glucosyl (1 → 2) β-D-galactoside 7-*O*-β-D glucoside, apigenin-6,8-di-*C*-rhamnosyl-glucosyl, vitexin, isovitexin, and 4,2′,4′-trihydroxy-7-methoxyisoflavan-4′-*O*-β-D-glucopyranoside, showed the highest binding affinities (Supplementary materials, Table S1). The glycone moieties in each of these compounds bound at the bottom of the pocket and established an array of hydrogen bonds with active site residues (Supplementary materials, Figure S1); 4,2′,4′-trihydroxy-7-methoxyisoflavan-4′-*O*-β-D-glucopyranoside showed hydrogen bond interactions with Asp616, His674, Asp404, and Asp518 amino acids (Figure 4a); kaempferol 3-*O*-β-D-glucosyl (1 → 2) β-D-galactoside 7-*O*-β-D glucoside exhibited hydrogen bonding with Asp616, Arg600, Asp282, and Gly651 residues (Figure 4b); quercetin-3,7-*O*-α-L di-rhamnoside interacted through hydrogen bonds with Asp282, Asp616, Asp518, Asp404, and His674 (Figure 4c); and apigenin-6,8-di-*C*-rhamnosyl-glucosyl revealed hydrogen bond interactions with Asp518, Asp404, His674, and Asp282 (Figure 4d), while the aglycone moieties located at the entrance of the active site engaged in only few interactions (Figure 4). All these interactions helped the compounds to anchor in the active site of α-glucosidase. To the best of our knowledge, several flavonoids have been reported to be effective as α-glucosidase inhibitors [51,52,53,54]. The isoflavan 4,2′,4′-trihydroxy-7-methoxyisoflavan-4′-*O*-β-D-glucopyranoside has been previously isolated from *T. stellata* and reported to have antidiabetic activity through increasing the activity of PPARα and PPARγ receptors as well [20]. However, our results indicated that this isoflavan compound inserted itself into the active site of the α-glucosidase enzyme, showed a high binding affinity, and is involved in an array of hydrogen bonding interactions with active site residues, which could be an additional mechanism of its antidiabetic activity. On the other hand, the amino acid (4-hydroxyleucine), detected in the LC-HRESIMS results, displayed weak interactions with the α-glucosidase enzyme; however, previous research reported that it has an important role controlling blood sugar levels through the regulation of insulin secretion in both animals and humans [55,56]. In conclusion, the α-glucosidase inhibition assay and docking study shed light on *T. stellata* as a promising antidiabetic plant, which needs more detailed future investigation.

## 3. Material and Methods

### 3.1. Plant Material

Aerial parts of *T. stellata* were collected from the Mediterranean coast of Egypt in March 2017; the plant was kindly identified by Dr. Ibrahim El-Garf, Faculty of Science, Cairo University and Dr. Abdel Haleem Mohamed, Flora and Phytotaxonomy Department, Agricultural Research Center. A voucher specimen (BUPD-81-2017) was deposited at the Pharmacognosy Department Herbarium, Faculty of Pharmacy, Beni-Suef University. The plant material was air-dried and grounded into powder. The grinded plant was maintained in an airtight container and kept for further analysis.

### 3.2. Chemicals

Methanol (MeOH) was purchased from El-Nasr Company for Pharmaceuticals and Chemicals (Cairo, Egypt). Solvents for High-Performance Liquid Chromatography (HPLC) were purchased from Sigma-Aldrich (Saint Louis, MO, USA), including HPLC-methanol and HPLC-water. For the biological study, DPPH, rutin, and ascorbic acid were purchased from (Sigma-Aldrich, Saint Louis, MO, USA).

### 3.3. Extraction

One gram of the plant powder was extracted using 50 mL of methanol (80%) for 2 h at room temperature on an orbital shaker adjusted at 200 rpm. After centrifugation for 20 min, the supernatant was transferred to a 100 mL volumetric flask. The procedure was repeated, and the collective supernatant volume was adjusted to 100 mL and used for total phenolic and total flavonoid assays [57].

### 3.4. Total Phenolic and Total Flavonoid Content

The total phenolic content was measured using Folin–Ciocalteu reagent [58]. An amount of 300 µL of the extract was added to 2.25 mL of Folin–Ciocalteu reagent and allowed to stand for 5 min at room temperature, and then 2.25 mL of sodium carbonate solution (60 g/L) was added to the mixture and incubated for 90 min at room temperature. Then, the absorbance of the developed color was measured at 725 nm. Gallic acid was used to prepare a standard curve for quantitative purposes, where results were calculated as µg of gallic acid equal to 1 g of the dried sample, while the total flavonoid was evaluated according to method of [29]. In a test tube, 0.5 mL of the alcoholic extract was mixed with 2.25 mL of distilled water and 0.15 mL of 5% NaNO_2_ solution. The mixture was vortexed and allowed to stand for 6 min, and then 0.3 mL of AlCl_3_·6H_2_O solution (10%) was added. After 5 min, it was followed by adding 1.0 mL of 1 M NaOH and mixed well using vortex, and then the absorbance was immediately measured at 510 nm. Results were expressed as mg rutin equivalents for 1 g of dried sample (mg RE/g).

### 3.5. Biological Activity

#### 3.5.1. Antioxidant Activity

The antioxidant activity of the *T. stellata* extract was evaluated using 2,2-diphenyl-1-picrylhydrazyl (DPPH) free radical scavenging [59]. An aliquot of 300 µL of sample (1000–250 µg/mL) with 3.0 mL of DPPH in methanol (60 µg/mL) were mixed and allowed to stand for 30 min in the dark at room temperature. Then, the absorbance was measured at 517 nm, using ascorbic acid as the positive control. The free radical scavenging activity was calculated using the following equation: Scavenging effect (%) = [1 − (absorbance of sample/absorbance of control)] × 100.

#### 3.5.2. Antidiabetic Activity

α-Glucosidase inhibitor Screening Kit (Colorimetric) (Catalog # K938-100) and α-Amylase Inhibitor Screening Kit (Catalog # K482-100) were purchased from Biovision. Acarbose was used as the positive control in both assays.

The α-glucosidase inhibition assay depends on the ability of an active α-glucosidase to cleave a synthetic substrate, thus releasing a chromophore (λ_max_ 410 nm). In the presence of an α-glucosidase-specific inhibitor, the enzymatic activity is greatly reduced, which is detected by a decrease in absorbance readings. An α-glucosidase inhibition assay was performed following a previously described method with slight modifications [60], and *p*-nitrophenyl-α-D-glucopyranoside (PNPG) was used as the substrate. A mixture of 10 µL of extract, 130 µL of phosphate buffer (pH 6.5, 30 mM), and 10 µL of enzyme was pre-incubated at room temperature for 5 min before 50 µL of PNPG substrate solution (10 mM) was added, and the reaction mixture was further incubated at room temperature for 15 min. The reaction was quenched by the addition of 50 µL of 2 M of glycine (pH 10) into the mixture. The absorbance of the liberated *p*-nitrophenol was measured at 410 nm.

Human α-amylase was used to hydrolyze the synthetic substrate, yielding smaller fragments containing the chromophore (λ_max_ = 405 nm). *T. stellata* extract (1 mL) was mixed with 1% α-amylase enzyme and incubated for 10 min. Soluble starch (1 mL of 1% starch in phosphate buffer pH 7.4) was added to the mixture and further incubated for 10 min at 25 °C. The reaction was stopped by adding 1 mL of 1% dinitrosalicylic acid and boiled at 90 °C in a water bath for 15 min. The cooled reaction mixture was diluted with 1 mL of de-ionized water and the absorbance was measured at 405 nm at 20 °C [61]. 

#### 3.5.3. Cytotoxic Activity

Cytotoxic activity was measured through the tissue culture center in the Egyptian Organization for Biological Products and Vaccines, Vacsera, Egypt. Three cell lines were selected for the assay: liver carcinoma cell line (Hep-G2, Accession number HB-8065), breast carcinoma cell line (MCF-7, Accession number HTB-22), and intestinal carcinoma cell line (Caco-2, Accession number HTB-37). Tested cell lines were obtained from the American Type Culture Collection (ATCC, Rockville, MD, USA). Doxorubicin was used as the positive control. DMEM, RPMI-1640, fetal bovine serum, HEPES buffer solution, gentamicin, L-glutamine, and 0.25% Trypsin-EDTA were obtained from Lonza and used for cytotoxicity measurement. The viability assay followed the method reported by Mosmann T. [62,63]. In brief, the cell lines were suspended in medium at a concentration 5 × 10^4^ cell/well in Corning^®^ 96-well tissue culture plates and then incubated for 24 h. The tested extracts were then added into 96-well plates (three replicates) to achieve twelve concentrations for each concentration. DMSO was used as the control, and after incubation, the numbers of viable cells were determined by MTT test.

The relation between surviving cells and drug concentration was plotted to obtain the survival curve of each tumor cell line after treatment with the extract. The IC_50_ value was defined as the concentration of the extract required to inhibit 50% of cell growth, and it was estimated from graphic plots of the dose–response curve for each concentration using GraphPad Prism software (San Diego, CA, USA).

#### 3.5.4. Statistical Analysis

All the results were expressed as mean values ± SE from three separate experiments. The IC_50_ values were calculated from the dose–response curves using nonlinear regression analysis that gave a percentage of the inhibition values. Group differences were determined by analysis of variance (ANOVA). When statistically significant differences were indicated by ANOVA, the values were compared by the Tukey test. The differences were considered statistically significant from the controls at *p* < 0.05.

### 3.6. Chemical Profiling

Metabolomic analysis of the crude alcoholic extract of *T. stellata* was performed using the LC-HRESIMS technique [64]. The analysis was performed on an Acquity Ultra Performance Liquid Chromatography system coupled to a Synapt G2 HDMS quadrupole time-of-flight hybrid mass spectrometer (Waters, Milford, MA, USA). Chromatographic separation was achieved on a C18 column (2.1 × 100 mm, particle size 1.7 µm; Waters, Milford, MA, USA) with a guard column (2.1 × 5 mm, particle size 1.7 µm) and a linear binary solvent gradient of 0–100% of eluent B over 6 min with a flow rate of 0.3 mL min^−1^. Solvent A was composed of 0.1% formic acid in water (*v*/*v*), while solvent B was acetonitrile. An amount of 2 µL of sample was injected, and the column temperature was set at 40 °C.

Detection of the metabolites was performed after chromatographic separation using mass spectrometry by electrospray ionization (ESI) in positive mode, and the source was set to 120 °C. The ESI capillary voltage was operated at 0.8 kV, while the sampling cone voltage was adjusted to 25 V. Nitrogen gas was used as the desolvation gas at a 800 L h^−1^ flow rate and 350 °C and the cone gas (flow rate of 30 L h^−1^). The mass range for TOF–MS was set to *m*/*z* 50–1200. The raw data were imported in MZmine 2.12 through selection of the ProteoWizard converted positive files in mzML format. Mass ion peaks were detected, followed by a chromatogram builder and chromatogram deconvolution. The local minimum search algorithm was applied, and the isotopes were identified via an isotopic peaks grouper. Detection of the missing peaks was performed using a gap-filling peak finder. An adduct search, in addition to a complex search, was applied. Then, the processed dataset was subjected to molecular formula prediction, as well as peak identification. The positive ionization mode datasets from plant extract were dereplicated against the Dictionary of Natural Products (DNP) database.

### 3.7. Molecular Docking

Docking studies were accomplished using the Molecular Operating Environment (MOE) (version 2015.10) as the computational software. The X-ray crystallographic structure of human lysosomal acid alpha-glucosidase co-crystallized with the ligand acarbose (PDB ID: 5NN8) was obtained from the Protein Data Bank https://www.rcsb.org/structure/5NN8 (accessed on 23 November 2021) to investigate binding affinities, modes, and interactions of compounds toward the α-glucosidase receptor site. Hydrogen atoms and missed bonds and connections were added, and the potential of the receptor atoms was fixed. To evaluate the accuracy of docking, a re-docking process of the co-crystallized ligand (acarbose) with human lysosomal acid α -glucosidase (PDB ID: 5NN8) was performed using the Amber12:EHT forcefield, London dG scoring function for the placement of poses, and the GBVI/WSA dG scoring function for poses refinement to detect the binding energy score, amino acid interactions, and relative mean square deviation (rmsd). The re-docking process succeeded in regenerating the orientation of the co-crystallized ligand with rmsd value of 1.1 Å. A virtual database of identified compounds in *T. stellata* was energy-minimized. The entire energy-minimized library was docked with the prepared catalytic domains of α-glucosidase using the same parameters as in the re-docking process. For each docked compound, 30 docked poses were chosen, followed by refinement into the best 5 docked poses. The binding interactions of the docked compounds with the receptor and docking score were studied using 2D and 3D pictures.

## 4. Conclusions

The present study represented a comprehensive analysis of the alcoholic extract of *Trigonella stellata*, as well as a biological assessment and molecular docking study. Metabolic profiling using LC-HRESIMS resulted in the dereplication of 15 compounds, including eleven compounds detected for the first time in the plant. In vitro testing of α-glucosidase inhibitory activity suggested the inhibition of carbohydrates-metabolizing enzymes as an additional mechanism for the antidiabetic potential of *T. stellata*. Reviewing the relevant literature for the reported α-glucosidase inhibitory activity of the resolved compounds, followed by virtual assessment using the molecular docking study, concluded that graecumoside A, fenugreekine, 4-hydroxyisoleucine, and the aforementioned six flavonoid glycosides might be responsible for the antidiabetic activity of *T. stellata* through different mechanisms such as α-glucosidase inhibitory activity, stimulation of PPARα and PPARγ receptor activity, as well as the regulation of insulin secretion. Hence, further phytochemical and biological investigations of this plant are recommended.

## Figures and Tables

**Table 1:** The LC-HRESIMS dereplication results of *Trigonella stellata* methanolic extract.

Metabolite Name	Molecular Formula	RT (min)	*m*/*z*	Plant Source	Reference
Rhapontigenin	C_15_H_14_O_4_ *	3.5879	258.1368	*T. foenum-graecum*	[47]
Gallic acid	C_7_H_6_O_5_	4.1712	171.0821	*T. foenum-graecum*	[45]
Kaempferol	C_15_H_10_O_6_ *	4.2564	285.2079	*T. foenum-graecum*	[45]
Diosgenin	C_27_H_42_O_3_	4.8606	414.2121	*T. foenum-graecum*	[46]
Isovitexin or Vitexin	C_21_H_20_O_10_	5.1357	432.2447	*T. foenum-graecum*	[45]
7,4′-dihydroxyflavone	C_15_H_10_O_4_	5.5667	254.1596	*T. stellata*	[20]
4-hydroxyisoleucine	C_6_H_13_NO_3_	6.1757	147.0239	*T. foenum-graecum*	[43]
4,2′,4′-trihydroxy-7-methoxyisoflavan-4′-*O*-β-D-glucopyranoside	C_22_H_26_O_10_	6.2080	450.3261	*T. stellata*	[20]
Demethylmedicarpin	C_15_H_12_O_4_ *	6.2163	256.1580	*T. foenum-graecum*	[44]
Graecumoside A	C_27_H_38_O_11_	6.2318	538.8999	*T. foenum-graecum*	[42]
Quercetin3,7-*O*-α-L dirhamnoside	C_27_H_30_O_15_	6.3316	593.9521	*T. stellata*	[20]
(2R,3S,4R)-4,2′,4′-trihydroxy-2,7dimethoxy isoflavan	C_17_H_18_O_6_	6.6067	318.2840	*T. stellata*	[20]
Fenugreekine	C_21_H_27_N_7_O_14_P_2_	6.7443	663.3963	*T. foenum-graecum*	[45]
Kaempferol 3-*O*-β-D-glucosyl (1 → 2) β-D-galactoside 7-*O*-β-D glucoside	C_33_H_41_O_23_	6.7960	771.6169	*T. foenum-graecum*	[48]
Apigenin-6,8-di-*C*-rhamnosyl-glucosyl	C_21_H_30_O_14_	8.9940	577.2966	*T. foenum-graecum*	[49]

* These compounds were annotated as sodium ion adduct [M + Na]^+^.

## Data Availability

Data is contained within the article and Supplementary Materials.

## Supplementary Materials

The following are available online at https://www.mdpi.com/article/10.3390/plants11020208/s1, Figure S1: 2D diagram of the binding interactions of compounds detected in *Trigonella stellata* to α-glucosidase enzyme (PDB: 5NN8) binding site, Table S1: Molecular docking data of compounds detected in *Trigonella stellata* against α-glucosidase enzyme (PDB: 5NN8).
