# Supplementary material for: Characterization of Possible α-Glucosidase Inhibitors from Trigonella stellata Extract Using LC–MS and In Silico Molecular Docking
Source: Plants (Basel). 2022 Jan 14;11(2):208. doi: 10.3390/plants11020208 (PMC8780848; doi:10.3390/plants11020208)
Supplement: Supplementary file 1 [file plants-11-00208-s001.zip › plants-1532243-supplementary.pdf]

Characterization of Possible α-Glucosidase Inhibitors from *Trigonella stellata* Extract Using LC-MS and In-Silico Molecular Docking

(SUPPLEMENTARY MATERIAL)

Ahlam Elwekeel[1†], Dalia El Amir[1]†, Enas I. A. Mohamed[1], Elham Amin[1,2], Marwa H. A. Hassan[1]* and Mohamed A. Zaki[1]*

[1]Department of Pharmacognosy, Faculty of Pharmacy, Beni-Suef University, Beni-Suef 62514, Egypt.

[2]Department of Medicinal Chemistry and Pharmacognosy, College of Pharmacy, Qassim University, Buraidah 52571, Saudi Arabia

*Corresponding authors

[†]Authors with equal contributions

Figure S1: 2D diagram of the binding interactions of compounds detected in Trigonella stellata to α-glucosidase enzyme (PDB: 5NN8) binding site.

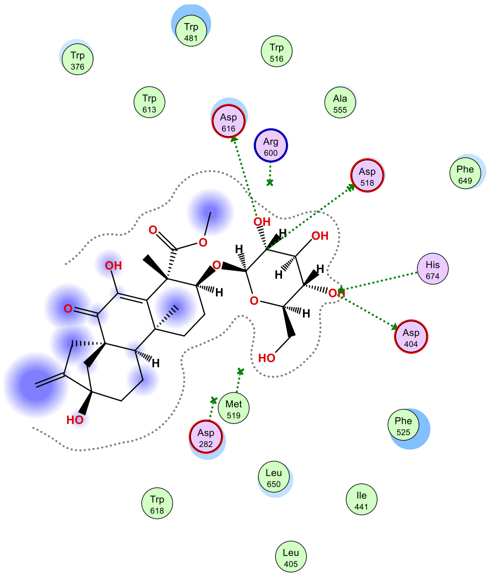

Graecumoside A

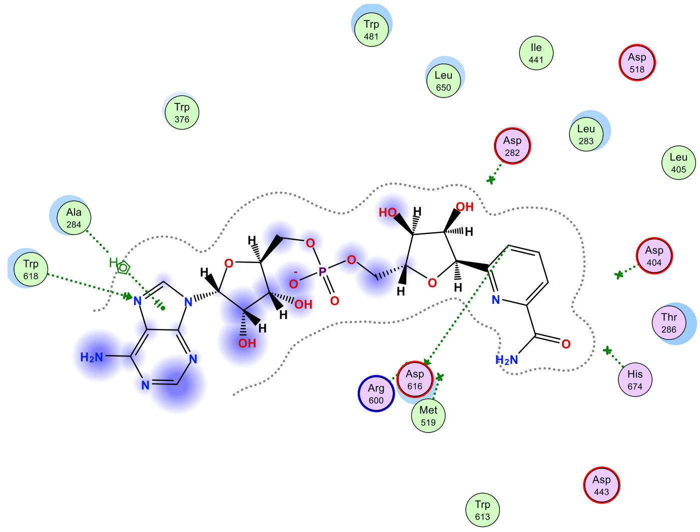

Fenugreekine

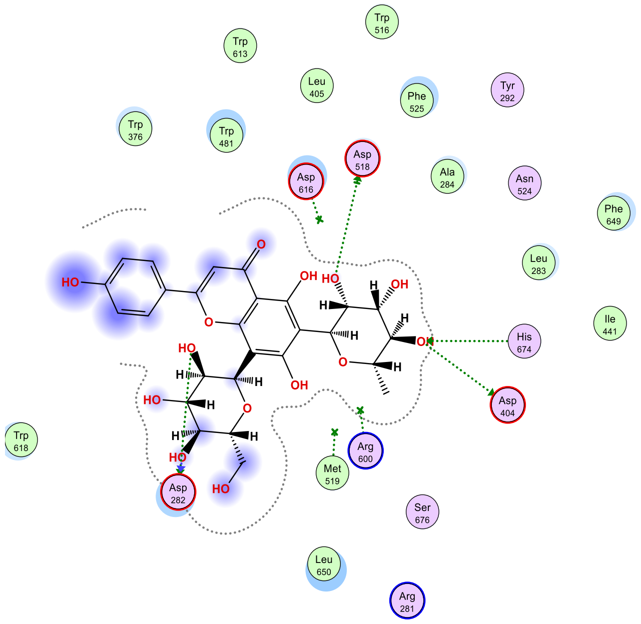

Apigenin-6,8-di-*C*-rhamnosyl-glucosyl

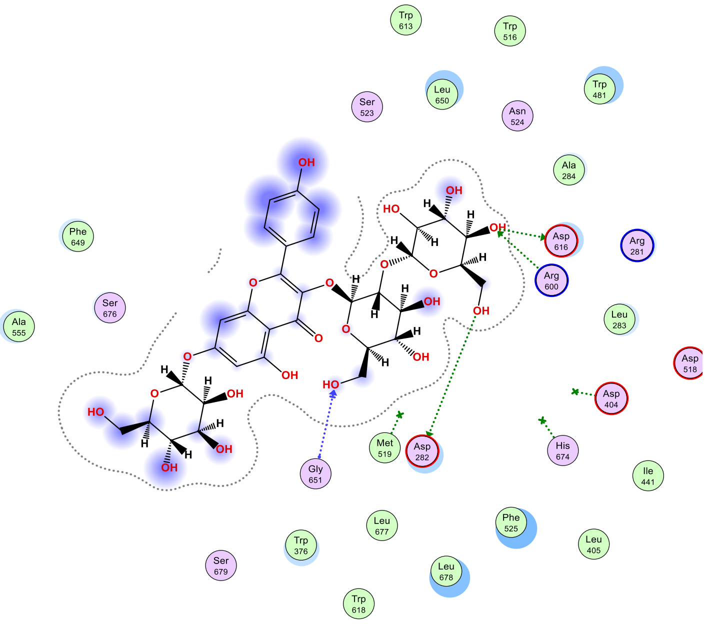

Kaempferol 3-O-*β*-D-glucosyl (1→2) *β*-D-galactoside 7-*O*-β-D glucoside

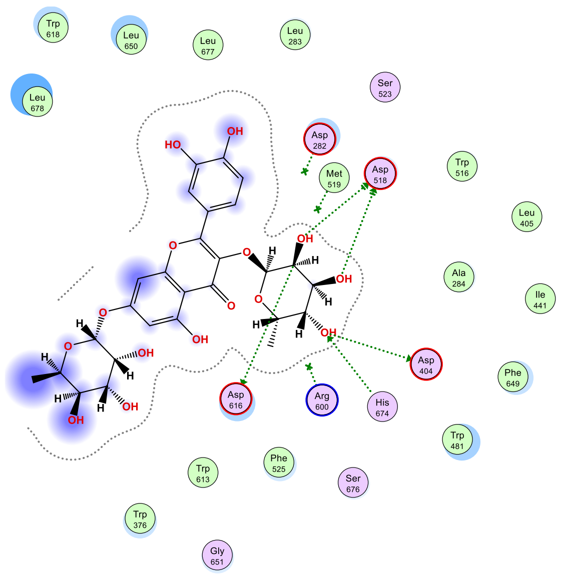

Quercetin3,7-*O*-*α*-L dirhamnoside

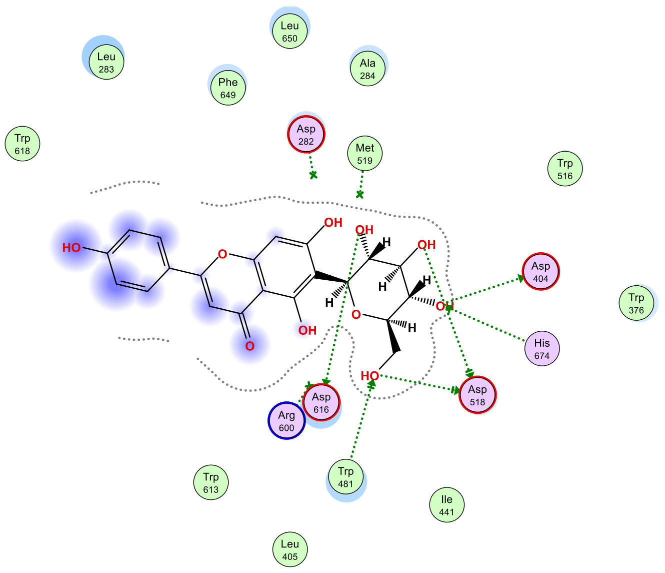

Isovitexin

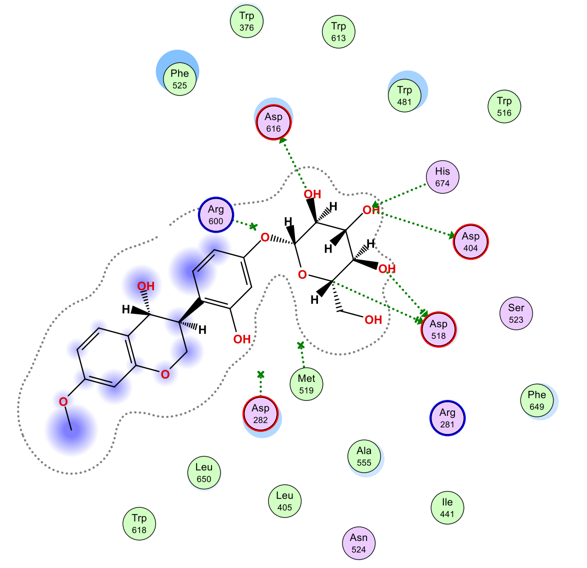

4,2′,4′-trihydroxy-7-methoxyisoflavan-4′-*O*-*β*-D-glucopyranoside

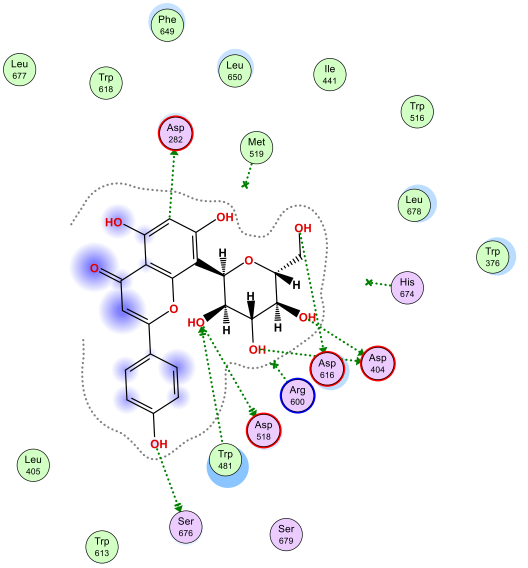

Vitexin

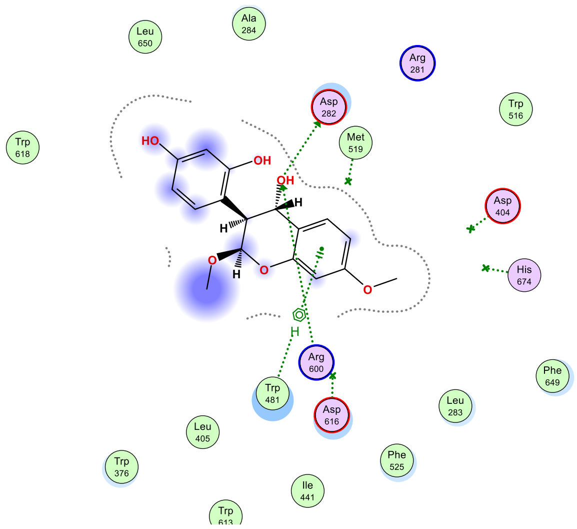

4,2′,4′-trihydroxy-2,7dimethoxy isoflavan

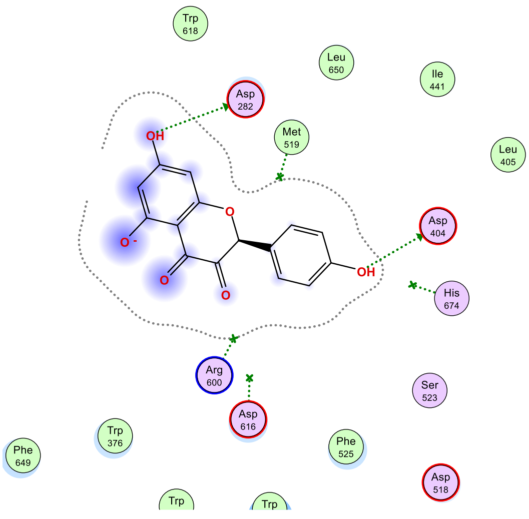

Kaempferol

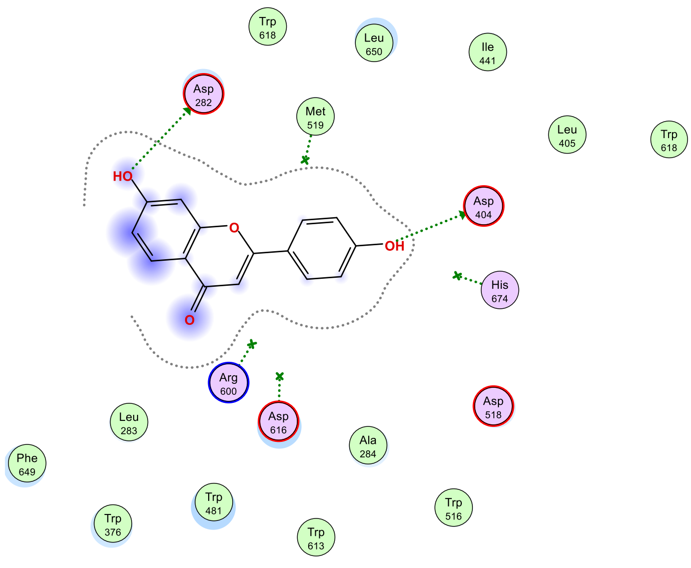

7,4′-dihydroxyflavone

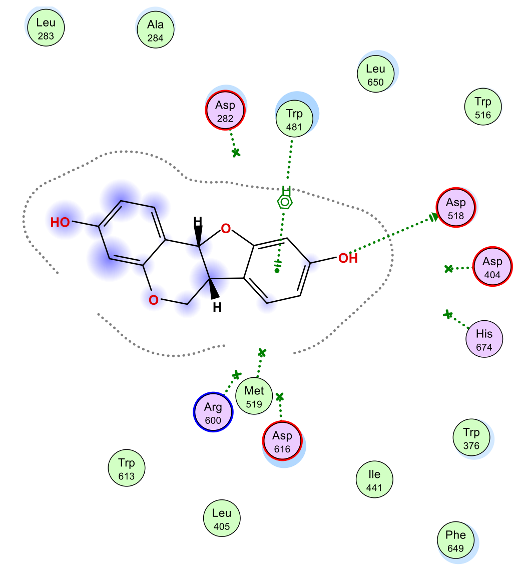

Demethylmedicarpen

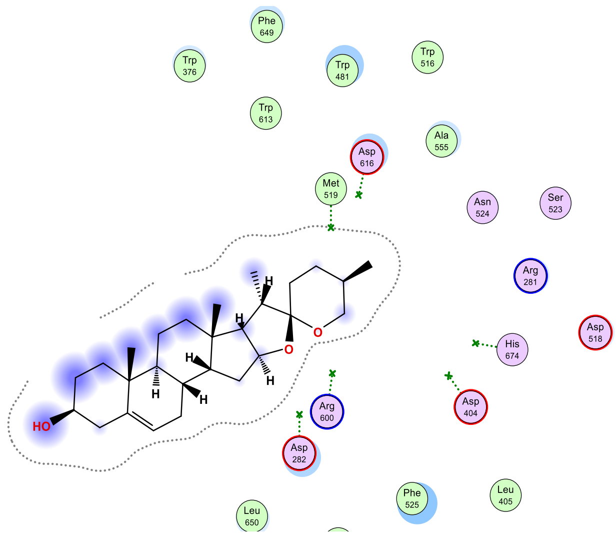

Diosgenin

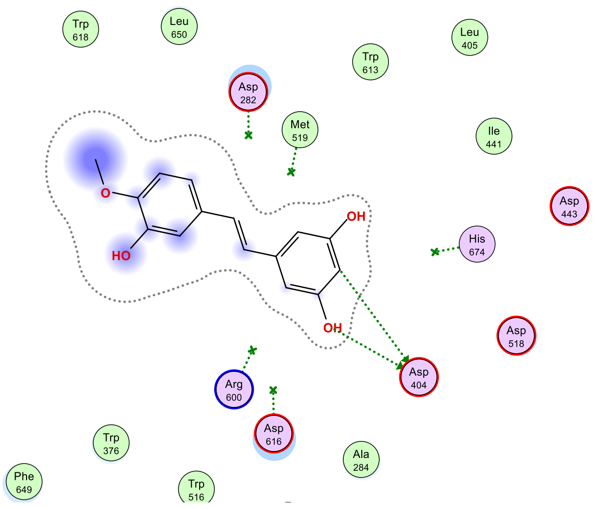

Rhapontigenin

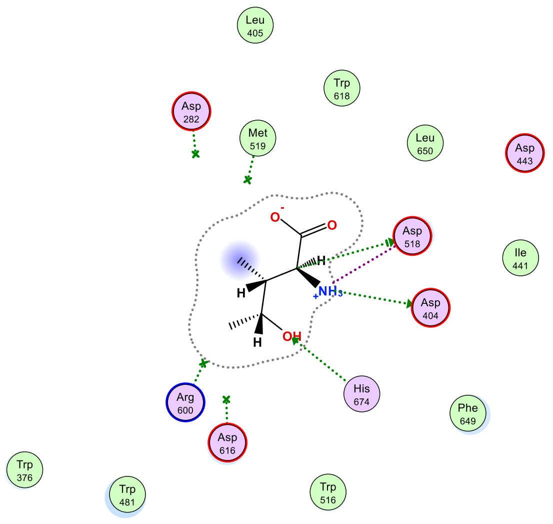

4-Hydroxyisoleucine

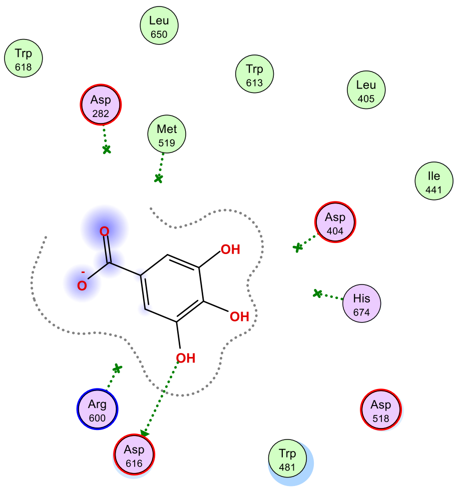

Gallic acid

Table S1: Molecular docking data of compounds detected in *Trigonella stellata* against α-glucosidase enzyme (PDB: 5NN8).

Compound	S	E_place	E_refine
Quercetin3,7-O-α-L dirhamnoside	-8.4145	-122.8735	-56.2776
Kaempferol 3-O-β-D-glucosyl (1→2) β-D-galactoside 7-O-β-D glucoside	-8.2965	-124.818	-50.5163
Apigenin-6,8-di-*C*-rhamnosyl-glucosyl	-7.9316	-70.4017	-48.7354
4,2′,4′-trihydroxy-7-methoxyisoflavan-4′-*O*-*β*-D-glucopyranoside	-7.5373	-79.0674	-43.2596
Graecumoside A	-7.5332	-79.6454	-44.9570
Fenugreekine	-7.4232	-67.1776	-43.5502
Isovitexin	-7.6797	-81.5558	-53.8580
Vitexin	-7.3030	-76.0586	-37.0603
Diosgenin	-5.7770	-50.8094	-25.5028
4,2′,4′-trihydroxy-2,7dimethoxy isoflavan	-5.3993	-56.7509	-26.3511
Kaempferol	-5.1935	-49.4873	-20.8675
4-Hydroxyisoleucine	-5.1152	-50.5002	-22.1747
Rhapontigenin	-5.0388	-66.4366	-21.5091
7,4′-dihydroxyflavone	-4.8529	-87.7661	-20.3868
Demethylmedicarpin	-4.8493	-62.5706	-22.8222
Gallic acid	-4.2908	-57.5793	-14.8477
